# Evaluation of Secondary Bacterial Infections and Determination of Antibiogram Susceptibility Testing in Hospitalized Patients with COVID-19

**DOI:** 10.30699/IJP.2024.2006587.3141

**Published:** 2024-10-02

**Authors:** Tahmineh Mollasharifi, Mahmoud Zamani, Atoosa Gharib

**Affiliations:** 1 *Department of Pathology, Clinical Research Development Center, Shahid Modarres Educational Hospital, Shahid Beheshti University of Medical Sciences, Tehran, Iran*; 2 *Department of Pathology, Shahid Beheshti University of Medical Sciences, Tehran, Iran*

**Keywords:** COVID-19, Coinfection, Drug resistance, Intensive care unit

## Abstract

**Background & Objective:**

COVID-19 is a global pandemic that has caused an increase in hospitalization rates and high mortality. Secondary bacterial infections in hospitalized patients are one of the common complications of this viral disease. Due to the increased prevalence of antibiotic resistance, treating these patients is challenging. Therefore, this study aimed to evaluate the secondary bacterial infection and antimicrobial sensitivity test in hospitalized patients with COVID-19 in a tertiary hospital.

**Methods:**

In this retrospective descriptive study, all patients with COVID-19 who were admitted to Shahid Modares Hospital (Tehran-Iran) from October 2020 to March 2021 with positive culture results for bacterial infections, were assessed. The significance level was lower than 0.05.

**Results:**

Ninety-seven patients with a mean age of 65.23 ± 16.72 years were assessed. The male patients accounted for 58.8% of the patients, while 41.2% were female. The ICU admitted patients with critical COVID-19 severity accounted for 59.8%, while 40.2% were hospitalized in the ward with a severe form of the disease. Age, length of hospitalization, and mortality rate were significantly higher in patients with ICU admission (all P-values<0.05). The most antibiotic-resistant bacteria were *Klebsiella pneumoniae* (32.98%). ICU admission showed a significantly higher rate in patients who were resistant to Cefotaxime, Ceftriaxone, Gentamicin, and Co-trimoxazole compared to the patients who were hospitalized in the ward (all P-values<0.05).

**Conclusion:**

Secondary bacterial infection in hospitalized patients with COVID-19 may lead to high mortality.

## Introduction

COVID-19 is an infectious disease caused by the SARS-CoV-2 virus that has infected millions of people worldwide and caused high mortality in the world. Clinical symptoms of COVID-19 include a wide spectrum from asymptomatic infection to life-threatening pneumonia. Some patients, especially those with respiratory distress, are hospitalized, and severe cases are admitted to the ICU with mechanical ventilation support (1-3).

Bacterial co-infection is a concerning complication in viral respiratory infections (4). This complication worsens the patient’s condition, especially in ICU-admitted patients (5, 6). Although there are some reports about the prevalence, incidence, and mortality rate of secondary bacterial infection in the hospitalized patients with COVID-19, there is a need for more studies on the prevalence of secondary bacterial infection and antibiotic resistance in these patients (7, 8).

In the early phase of the pandemic, concerns about COVID-19, the unknown nature of the disease, the difficulty in rapidly eradicating bacterial infections, and the lack of treatment guidelines led physicians to a widespread use of antibiotics, both as directed and empiric therapy (9). In this phase, from December 2019 to June 2020, antibiotic prescriptions were reported in 82.3% of patients with COVID-19 (11). This was in line with several other studies that also documented increased initial use of antibiotics for COVID-19 patients and widespread use of empiric antibiotic therapy in the hospitalized COVID-19 patients (10-13).

Despite the administration of intensive antibiotic therapy, the mortality rate among patients with COVID-19 is high due to the secondary bacterial infection (13-15). COVID-19 patients who were hospitalized are at risk of secondary bacterial involvement with hospital-acquired multidrug-resistant (MDR) bacteria. Widespread use of intensive antibiotic therapy has caused an increase in the prevalence of drug-resistant bacteria (16-18). Studies have shown secondary microbial infections with MDR bacteria. Even azole-resistant Aspergillus has been reported in hospitalized COVID-19 patients (19). The aim of this study was evaluation of secondary bacterial infections and determination of antibiogram susceptibility testing in the hospitalized COVID-19 patients.

## Material and Methods

This retrospective descriptive study was conducted on all patients with COVID-19 who were admitted to Shahid Modares Hospital (Tehran-Iran) from October to March 2019. The inclusion criteria were hospitalization due to COVID-19 confirmed by polymerase chain reaction (PCR) test and presence of the signs and symptoms of secondary bacterial infection confirmed by a positive culture of a new pathogen after hospitalization. Exclusion criteria were having other infectious diseases before infection with SARS-COV-2 and an incomplete patient file.

According to the definition, secondary bacterial infection refers to patients who showed clinical signs and symptoms of bacterial infections. At least one case of a positive bacterial agent from their appropriate sample for microbiology examination (sputum, endotracheal aspirate, bronchoalveolar lavage fluid, blood, or urine sample) was obtained after SARS-COV-2 involvement and being hospitalized (20). Bacterial sensitivity and resistance were assessed according to CLSI 2020 standards and determined based on CLSI 2020 (21).

According to the severity of the disease during hospitalization, patients with shortness of breath, a respiratory rate more than 30 per minute, arterial blood oxygen saturation less than 93%, partial pressure of arterial oxygen and fraction of inspired oxygen ratio < 300, and pulmonary infiltrate more than 50% within 24 to 48 hours after hospitalization was classified as severe disease group (22). Patients who presented with respiratory failure, septic shock and dysfunction, or multiple organ failure were classified as very severe or critical disease groups (22). The electronic file information of the patients was checked, and their information was entered into the checklist. 

### Statistical Analysis

Mean, standard deviation, frequency, and percentage were used to analyze the data. The Chi-square test and Fisher Exact test were used to compare qualitative variables between the two groups. T-test was also used for quantitative variables. All analyses were performed by SPSS 26.0 statistical software (SPSS Inc., Chicago, Ill., USA). A P-value less than 0.05 was considered statistically significant.

## Results

After the assessment of 1590 hospitalized patients, the records of 97 patients were included in the study. Fifty-eight patients (59.8%) were hospitalized in the ICU with a very severe or critical disease type (critical group), and 39 patients (40.2%) were hospitalized with a severe disease type (severe group). The mean age of the patients who were administered in ICU was 68.16±16.07 years, and in patients who were administered in the ward with a severe form of COVID-19, it was 60.87±16.91 years (*P*=0.035). 

The data about age, sex, duration of hospitalization, co-infection type, and outcome are shown in Table 1. Our findings showed significant age and hospitalization duration differences between patients with severe form and critical form (*P*<0.05). 

In Table 2, we examined the prevalence of the bacteria in two Gram-negative and Gram-positive subtypes based on the specimens. In total, in 30 cases, bacteria were obtained from blood cultures (30.9%), 35 cases from urine cultures (36.1%), 18 cases from tracheal cultures (18.6%), and 14 cases from other cultures (14.4%). The related data is seen in Table 2.

**Table 1:** Demographic and clinical information and prognosis of the patients with secondary infection in the hospitalization course of COVID-19

		Total (97)	ICU (Critical) (58)	Ward (Severe) (39)	P-value	
Characteristics							
Age(year)	Mean ± SD	65.23± 16.72	68.16 ± 16.07	60.87 ± 16.91		0.035 *	
	Median (IQR)	68(44.77)	70.5(48.78)	61(44.72)			
Sex	Female	40 (41.2%)	27 (46.6%)	13 (33.3%)		0.214**	
	Male	57 (58.8%)	31 (53.4%)	26 (66.7%)			
Duration of hospitalization	Mean ± SD	12.56 ± 6.72	13.81 ± 7.58	10.69 ± 4.7		0.024 *	
Co-infection type	*Klebsiella pneumoniae*	32 (33.0%)	23 (39.7%)	9 (23.1%)		0.135	
	*Staphylococcus aureus*	12 (12.4%)	6 (10.3%)	6 (15.4%)		0.663	
	*Staphylococcus. epidermidis*	12 (12.4%)	7 (12.1%)	5 (12.8%)		0.918	
	Acinetobacter sp.	10 (10.3%)	8 (13.8%)	2 (5.1%)		0.294 **	
	*Pseudomonas. aeruginosa*	10 (10.3%)	3 (5.2%)	7 (17.9%)		0.09	
	*Escherichia coli* (*E. coli*)	8 (8.2%)	4 (6.9%)	4 (10.3%)		0.816	
	Enterococcus	8 (8.2%)	4 (6.9%)	4 (10.3%)		0.816	
	Yeast	5 (5.2%)	3 (5.2%)	2 (5.1%)		0.992	
Outcomes	Discharge	54 (55.7%)	15 (25.9%)	39 (100.0%)		<0.001**	
	Death	43 (44.3%)	43 (74.1%)	0 (0.0%)		0.752	

*P-value based on T-test

**P-value based on Chi-square or Fisher Exact

**Table 2:** The prevalence of Gram-negative and Gram-positive bacteria based on the type of specimen

		Sample Type			
		B/C (30)	Urine (35)	Trachea (18)	Other (14)
Gram-positive					
Bacteria type	*Staphylococcus aureus*	6 (20.0%)	3 (8.6%)	0 (0.0%)	3 (21.4%)
	*Staphylococcus epidermidis*	11 (36.7%)	1 (2.9%)	0 (0.0%)	0 (0.0%)
	Enterococcus	0 (0.0%	5 (14.3%)	0 (0.0%)	2 (14.3%)
	Yeast	1 (3.3%)	4 (11.4%)	0 (0.0%)	0 (0.0%)
Gram-negative					
Bacteria type	*Acinetobacter sp.*	2 (6.7%)	1 (2.9%)	6 (33.3%)	1 (7.1%)
	*E. coli*	1 (3.3%)	7 (20.0%)	0 (0.0%)	0 (0.0%)
	*Klebsiella. pneumoniae*	2 (6.7%)	14 (40.0%)	9 (50.0%)	7 (50.0%)
	*Pseudomonas aeruginosa*	7 (23.3%)	0 (0.0%)	2 (11.1%)	1 (7.1%)
	*Enterobacter sp.*	0(0.0%)	0(0.0%)	1(5.5%)	0(0.0%)

In Tables 3 and 4, the antimicrobial resistance of Gram-positive and Gram-negative bacteria was assessed. 

In Table 5, we assessed laboratory data between the two groups of hospitalized in the ward and the ICU admitted. The mean white blood cell (WBC) was significantly higher in the ICU-admitted patients (*P*<0.001), but there were no statistically significant differences in other variables between the two groups. 

In Table 6, we assessed level of the antimicrobial resistance in patients admitted to the ward and ICU. The amount of antibiotic resistance to Gentamicin, Co-trimoxazole (CTX), Ceftriaxone, and Cefotaxime was significantly higher in patients hospitalized in ICU (all P-values<0.05).

**Table 3:** Antimicrobial drug resistance in Gram-negative bacteria

Antimicrobial agent	Acinetobacter sp. (10)	*E. coli* (8)	*Klebsiella pneunoniae* (32)	*Pseudomonas aeruginosa* (10)
Tazocin	6(100.0%)	1 (100.0%)	12 (100.0%)	3 (100.0%)
Imipenem	5 (100.0%)	6 (75.0%)	8 (100.0%)	8 (100.0%)
Gentamicin	10 (100.0%)	5 (62.5%)	28 (89.6%)	7 (70.0%)
Co-trimoxazole (CTX)	10 (100.0%)	6 (75.0%)	30 (96.0%)	7 (70.0%)
Furadantin	1 (100.0%)	5 (62.5%)	30 (96.0%)	0 (0.0%)
Ciprofloxacin	10 (100.0%)	6 (75.0%)	26 (83.2%)	9 (90.0%)
Amikacin	8 (80.0%)	4(50.0%)	23 (73.6%)	7 (70.0%)
Ceftriaxone	10 (100.0%)	4(50.0%)	27(86.4%)	10 (100.0%)
Ceftazidime	7 (100.0%)	0 (0.0%)	15 (100.0%)	5 (100.0%)
Cefepime	10 (100.0%)	6 (75.0%)	32 (100.0%)	7 (70.0%)
Ampicilin-Sullbactam	6(100.0%)	0 (0.0%)	14 (100.0%)	4 (100.0%)
Meropenem	4 (100.0%)	3 (60.0%)	16 (100.0%)	3 (100.0%)
Cefotaxime	10(100.0%)	3(37.5%)	32 (100.0%)	8 (80.0%)
Temocillin	1(100.0%)	0 (0.0%)	3 (100.0%)	0 (0.0%)
Colistin	-	-	-	2(100.0%)

**Table 4:** Antimicrobial drug resistance in Gram-positive bacteria

	Bacteria type		
Antimicrobial agent Total (32)	***Staphylococcus aureus*** ** (12)**	***Staphylococcus epidermidis*** ** (12)**	**Enterococcus (8)**
Meropenem	0 (0.0%)	0 (0.0%)	0 (0.0%)
Cefazolin	00 (0.0%)	9 (75.0%)	8(100%)
Erythromycin	8(66.6%)	12 (100%)	8 (100.0%)
Co-trimoxazole (CTX)	3 (42.9%)	9 (75.0%)	8 (100.0%)
Ampicillin	9 (75.0%)	12 (100%)	4 (50.0%)
Ciprofloxacin	6 (66.7%)	6 (50.0%)	8 (100.0%)

**Table 5:** Evaluation of laboratory data between the two groups hospitalized in the ward and ICU

	Hospitalization			
		ICU(Critical)	Ward (Severe)	P-value
C-reactive protein (CRP)	1+	13 (22.4%)	9 (23.1%)	0.519**
	2+	17 (29.3%)	17 (43.6%)	
	3+	15 (25.9%)	6 (15.4%)	
	Neg	11 (19.0%)	5 (12.8%)	
	W.P	2 (3.4%)	2 (5.1%)	
White blood cells (Normal range: 4000-11000)		8331.69 ± 7309.9	3629.04 ± 4077.9	<0.001*
Neutrophil% (40-60%)		81.05 ± 14.05	75.76 ± 13.29	0.067*
Lymphocyte% (20-40%)		13.51 ± 13.71	14.94 ± 9.9	0.576*
Erythrocyte sedimentation rate (ESR) (up to 20mm/h)		41.75 ± 33.84	51.23 ± 38.14	0.225*
D- dimer (up to 500 ng/mL)		2538.79 ± 2288.59	1467.88 ± 1643.59	0.049*
Creatinine (up to 1.3 mg/dL)		2 ± 1.72	2 ± 1.47	0.991*
Pro-Calcitonin (up to 0.5 ng/mL)		8.84 ± 16	1.56 ± 1.58	0.168*
Ferritin (12- 200 µg/mL)		578.12 ± 598.31	682.79 ± 937.87	0.578*
IL- 6 (up to 9 pg/mL)		253.4 ± 328.78	188.13 ± 398.26	0.681*
Troponin (up to 0.1 µg /L)		2.63 ± 11.45	0.21 ± 0.88	0.217*

*P-value based on T-test

**P-value based on Chi-square or Fisher Exact

W.P=weakly Positive

**Table 6:** Assessment of the level of antimicrobial resistance based on the department

	Hospitalization		
Percentage of resistance	ICU (Critical)	Ward (Severe)	P-value*
Tazocin	12 (100.0%)	3 (100.0%)	0.03
Vancomycin	0 (0.0%)	0 (0.0%)	0.294
Imipenem	13 (100.0%)	6 (85.7%)	0.35
Clindamycin	7 (77.8%)	8 (57.1%)	0.4
Gentamicin	33 (86.8%)	13 (61.9%)	0.047
Erythromycin	9 (81.8%)	10 (90.9%)	>0.999
Co-Trimoxazole (CTX)	42 (91.3%)	21 (67.7%)	0.014
Cefazolin	1 (50.0%)	1 (25.0%)	>0.999
Colistin	2 (100.0%)	0 (0.0%)	0.333
Furadantin	6 (54.5%)	5 (71.4%)	0.637
Ciprofloxacin	45 (91.8%)	23 (76.7%)	0.092
Amikacin	23 (63.9%)	10 (47.6%)	0.274
Ceftriaxone	36 (97.3%)	15 (78.9%)	0.041
Cefotaxime	37 (100.0%)	16 (84.2%)	0.035
Ceftazidime	22 (100.0%)	5 (71.4%)	0.052
Cefazolin	7 (77.8%)	5 (55.6%)	0.62
Cefepime	31 (96.9%)	13 (81.3%)	0.101
Ampicillin	8 (100.0%)	8 (80.0%)	0.477
Ampicilin-Sullbactam	18 (100.0%)	4 (100.0%)	0.045
Meropenem	19 (95.0%)	8 (80.0%)	0.251
Cefixime	1 (100.0%)	1 (100.0%)	>0.999
Temocillin	9 (100.0%)	1 (100.0%)	0.08

P-value based on Fisher’s exact test

## Discussion

The present study was conducted on COVID-19 hospitalized patients for six months to evaluate secondary bacterial infection and determine antimicrobial sensitivity tests. Ninety-seven patients were assessed, of which 58 (59.8%) were hospitalized in the ICU with critical disease, and the rest had severe disease. Patients hospitalized in the ICU were significantly older, and the mean duration of hospitalization in this group was significantly longer. Secondary gram-negative bacterial infections (Klebsiella and Acinetobacter, etc.) showed a higher prevalence than Gram-positive bacteria (Staphylococcus and Enterococcus) (61.8% vs. 38.2%). Among patients admitted to the ICU, 74.1% died, while all patients admitted to the ward were discharged in good general condition. The most common bacteria obtained from blood culture was *Staphylococcus epidermidis* (36.7%), in the urine culture was *Klebsiella pneumoniae* (40%), and in the trachea culture the most bacteria were *K. pneumoniae* (50%). Regarding antibiotic resistance, the lowest resistance was to vancomycin, but 100% resistance was seen in several antibiotics’ prescriptions, such as cefixime and tazosin. The only significant laboratory finding that was different between the two groups was the WBC count, which was higher in the ICU group. The rate of antibiotic resistance to gentamicin, cotrimoxazole, ceftriaxone, and cefotaxime was significantly higher in ICU-admitted patients. 

In the present study, 58.8% of the patients were men, and the rest were women. The statistical difference between the two groups was not significant. In various studies, it has also been seen that there is no gender predominance in COVID-19 patients with secondary bacterial infection, and gender is not associated with secondary bacterial infection (23-25).

In different studies, the rate of infection in different parts of the body was different. For example, Bahceci* et al.* mentioned that in their study, among 92 patients, 31 patients only had positive blood culture infections, and 23 patients only had respiratory tract infections. On the other hand, simultaneous infection of the circulatory system and the respiratory system was observed in 38 patients (26).

Pourajm* et al.* showed that out of the total number of positive samples tested for bacteria, 88.6% of respiratory tract samples obtained from intratracheal aspiration, sputum, and bronchoalveolar lavage (BAL), 6.8% of blood samples, 1.4% of urine samples, and 1.4% of wound samples (20). In our study, 36.1% of patients demonstrated a positive urine culture, and 30.9% had a positive blood culture. Also, in 18.6% of cases, bacteria were isolated from the trachea, and in 14.4%, bacteria were isolated from other places. The reason for the difference in the results of infection sites needs to be investigated in future studies.

In a study conducted by Li *et al.*, 6.8% had a secondary bacterial infection, and half of these patients (51 people) died during hospitalization. They found patients with critical type COVID-19 had a higher chance of secondary bacterial infection. Among the 159 isolated bacterial strains, 136 strains (85.5%) were Gram-negative bacteria. *Acinetobacter baumannii* (35.8%), *K. pneumoniae* (30.8%), and *Stenotrophomonas maltophilia* (6.3%) were the most common bacteria. The drug-resistant rate to carbapenem was 91.2% and 75.5% in *A. baumannii* and *K. pneumonia*, respectively. Methicillin resistance was presented in 100% of *Staphylococcus aureus* and coagulase-negative Staphylococcus cases. Vancomycin resistance was not found (24). In the current study, it was seen that the most common bacteria were *K. pneumoniae* in 32 cases (33%), *S. aureus* in 12 cases (12.4%), *S. epidermidis* in 12 cases (12.4%), and *Pseudomonas aeruginosa* in 10 cases (10.3%), 10 cases (10.3%) of Acinetobacter sp., 8 cases (8.2%) of *Escherichia coli*, 8 cases (8.2%) of Enterococcus sp., and finally 5 cases (5.2%) of Yeast. In the current study, the prevalence of Gram-negative bacteria was about (61.8%) in general. The findings of these two studies were different from each other in terms of the prevalence of bacteria. In the current study, the mortality rate was directly related to the severity of the disease and hospitalization in the intensive care unit, which was similar to the study by Li* et al.* The lowest level of resistance in the gram-positive group was related to vancomycin (0.0%), which was similar to the study by Li* et al.* in which no resistance to vancomycin was seen. Since no resistance to vancomycin has been seen in the gram-positive group, vancomycin is recommended as a choice antibiotic for empiric therapy of gram-positive in secondary bacterial infection in COVID-19 patients. In the current study, it was seen that most of the bacterial species causing secondary bacterial infection were gram-negative. This finding was similar to the findings of previous studies conducted on secondary infection in COVID-19. In other studies, it has been seen that the most bacteria causing secondary infection in COVID-19 were gram-negative bacteria (3, 25, 27). 

In the study of Bahceci* et al.*, 1055 patients with COVID-19 were examined, and the results showed that 92 patients (8.7%) had proven bacterial infections of the respiratory system or blood. 64.1% of the mentioned patients were men. Among the microorganisms grown in blood culture, coagulase-negative staphylococcus, with a prevalence of 31%, and A. baumannii, with 27.5%, were more common. In respiratory tract cultures, *A. baumannii* was the most common, with a prevalence of 33.3%, followed by S. aureus and K. pneumoniae, each with 9.5%. The antibiotic-resistant bacteria was *A. baumannii*, which was resistant to all antibiotics except colistin. In the mentioned study, the use of empiric antibiotics was observed with a relatively high frequency (26). In the present study, it was seen that 53.4% of the patients were male; the most common bacteria isolated from the blood culture was *S. epidermidis*. It was also seen that the total number of *S. aureus* and *S. pidermidis* caused positive blood cultures in 56.7% of the cases, which is more frequent than the result of Bahceci *et al.*’s study. In the present study, the most common bacteria found in the respiratory tract were *K. pneumoniae* (50%) and Acinetobacter (33.3%), respectively, which is different from the study of Bahceci* et al.* In the current study, Klebsiella was the most antibiotic-resistant bacteria, which was different from the study of Bahceci* et al.* The difference in the results of the two studies can be related to the difference in sample size and geographical status of the two studies; however, further investigation would be required. 

In the study of Pourajm* et al.*, adults with severe COVID-19 were examined. Secondary bacterial infection was observed in 65 patients (11.9% of all patients hospitalized in the ICU). The mean age was 69.4 (range 21-95) years. Forty-two patients (63.6%) were men. The most common causes of secondary bacterial infection were *K. pneumoniae* (44 people) and *A. baumannii* (33 people). Most patients with secondary bacterial infection showed widespread drug resistance. The mortality rate among patients with secondary infection was 83%. The findings showed that *K. pneumoniae* and *A. baumannii* were the most common gram-negative bacilli resistant to carbapenem in COVID-19 patients admitted to the ICU. These findings emphasized the importance of strict implementation of infection control interventions and highlighted the role of antimicrobial surveillance during an epidemic (20). Klebsiella was noted as the most antibiotic-resistant bacteria in this study, similar to the current study. Klebsiella was the most common secondary bacterial infection after contracting COVID-19, and in this respect, the two studies showed similar results. Among the differences between the results of the present study and the study of Pourajam* et al.*, was that Acinetobacter was the second most common bacteria, but in the current study, the second most common bacteria was staphylococci (20).

Karatas *et al.*'s study showed that A. baumannii is the main respiratory pathogen in COVID-19 patients (9.76%). It was concluded in the patients with COVID-19, extended-spectrum beta-lactamase-producing Enterobacteriaceae are associated with a lower prevalence, and multidrug-resistant (MDR) *A. baumannii* had a higher prevalence in these patients (28). In the present study, it was seen that Klebsiella is the most common bacterial respiratory infection of COVID-19 patients, followed by Acinetobacter, and the two studies were different in this respect. This difference may also be due to the difference in the geographical area of the two studies and the difference in the prevalence of bacterial pathogens in the two areas, which, of course, need to be investigated in future studies.

## Conclusion

It is concluded that the most common and resistant pathogen causing secondary bacterial infection in patients with COVID-19 would be K.* pneumoniae* bacteria (32.98%), followed by staphylococcus (sum of *S. aureus* and *S. epidermidis* 24.48%). Duration of hospitalization and the mortality rate in the ICU-admitted patients may be significantly higher than in patients hospitalized in the ward. This issue can be due to the older age and the clinical condition of ICU-admitted patients. ICU-admitted patients may be significantly more resistant to cefotaxime, ceftriaxone, gentamicin, and cotrimoxazole than patients hospitalized in the ward. If a patient is involved with secondary bacterial infection during COVID-19 hospitalization, Vancomycin can be a choice due to no drug resistance against this antibiotic.

The relationship between antibiotic resistance and the final status of the patients needs to be investigated in further studies with a larger statistical population.

## Limitation

This single-center study was conducted at Shahid Modares Hospital in Tehran, Iran. Bacterial drug resistance may differ in other centers or regions, and these results should also be investigated in other centers. Mechanisms of bacterial resistance were not investigated in this study, and our information regarding the therapeutic effects of secondary bacterial infections was insufficient and should be investigated in future studies. 

## References

[1] Dong E, Du H, Gardner L (2020). An interactive web-based dashboard to track COVID-19 in real time. Lancet Infect Dis.

[2] Ripa M, Galli L, Poli A, Oltolini C, Spagnuolo V, Mastrangelo A (2021). Secondary infections in patients hospitalized with COVID-19: incidence and predictive factors. Clin Microbiol Infect.

[3] Sharifipour E, Shams S, Esmkhani M, Khodadadi J, Fotouhi-Ardakani R, Koohpaei A (2020). Evaluation of bacterial co-infections of the respiratory tract in COVID-19 patients admitted to ICU. BMC Infect Dis.

[4] Klein EY, Monteforte B, Gupta A, Jiang W, May L, Hsieh YH (2016). The frequency of influenza and bacterial coinfection: a systematic review and meta‐analysis. Influ Other Respir Viruses.

[5] Alhazzani W, Evans L, Alshamsi F, Møller MH, Ostermann M, Prescott HC (2021). Surviving sepsis campaign guidelines on the management of adults with coronavirus disease 2019 (COVID-19) in the ICU: first update. Crit Care Med.

[6] Organization WH (2020). Evidence to recommendations: methods used for assessing health equity and human rights considerations in COVID-19 and aviation: interim guidance, 23 December 2020.

[7] Cox MJ, Loman N, Bogaert D, O'Grady J (2020). Co-infections: potentially lethal and unexplored in COVID-19. The Lancet Microbe.

[8] Langford BJ, So M, Raybardhan S, Leung V, Westwood D, MacFadden DR (2020). Bacterial co-infection and secondary infection in patients with COVID-19: a living rapid review and meta-analysis. Clin Microbiol Infect.

[9] Goetz M, Graber C, Jones M (2020). Antibiotic use at veterans affairs' hospitals increases during COVID-19 pandemic, reversing a four-year downward trend.

[10] Baghdadi JD, Coffey K, Adediran T, Goodman KE, Pineles L, Magder LS (2021). Antibiotic use and bacterial infection among inpatients in the first wave of COVID-19: a retrospective cohort study of 64,691 patients. Antimicrob Agents Chemother.

[11] Goncalves Mendes Neto A, Lo KB, Wattoo A, Salacup G, Pelayo J, DeJoy III R (2021). Bacterial infections and patterns of antibiotic use in patients with COVID‐19. J Med Virol.

[12] Rawson TM, Moore LS, Zhu N, Ranganathan N, Skolimowska K, Gilchrist M (2020). Bacterial and fungal coinfection in individuals with coronavirus: a rapid review to support COVID-19 antimicrobial prescribing. Clin Infect Dis.

[13] Russell CD, Fairfield CJ, Drake TM, Turtle L, Seaton RA, Wootton DG (2021). Co-infections, secondary infections, and antimicrobial use in patients hospitalised with COVID-19 during the first pandemic wave from the ISARIC WHO CCP-UK study: a multicentre, prospective cohort study. Lancet Microbe.

[14] Feng Y, Ling Y, Bai T, Xie Y, Huang J, Li J (2020). COVID-19 with different severities: a multicenter study of clinical features. Am J Respir Crit Care Med.

[15] Zhou F, Yu T, Du R, Fan G, Liu Y, Liu Z (2020). Clinical course and risk factors for mortality of adult inpatients with COVID-19 in Wuhan, China: a retrospective cohort study. Lancet.

[16] Budinger GS, Misharin AV, Ridge KM, Singer BD, Wunderink RG (2021). Distinctive features of severe SARS-CoV-2 pneumonia. The Journal of clinical investigation.

[17] Lai C-C, Chen S-Y, Ko W-C, Hsueh P-R (2021). Increased antimicrobial resistance during the COVID-19 pandemic. International journal of antimicrobial agents.

[18] Hsu J (2020). How covid-19 is accelerating the threat of antimicrobial resistance. Bmj.

[19] Monnet DL, Harbarth S ( 2020). Will coronavirus disease (COVID-19) have an impact on antimicrobial resistance?.

[20] Pourajam S, Kalantari E, Talebzadeh H, Mellali H, Sami R, Soltaninejad F Secondary bacterial infection and clinical characteristics in patients with COVID-19 admitted to two intensive care units of an academic hospital in Iran during the first wave of the pandemic. Frontiers in cellular and infection microbiology.

[21] Weinstein MP, Lewis JS (2020). The clinical and laboratory standards institute subcommittee on antimicrobial susceptibility testing: background, organization, functions, and processes. J Clin Microbiol.

[22] Wu Z, McGoogan JM (2020). Characteristics of and important lessons from the coronavirus disease 2019 (COVID-19) outbreak in China: summary of a report of 72 314 cases from the Chinese Center for Disease Control and Prevention. Jama.

[23] Costa RLd, Lamas CdC, Simvoulidis LFN, Espanha CA, Moreira LPM, Bonancim RAB (2022). Secondary infections in a cohort of patients with COVID-19 admitted to an intensive care unit: impact of gram-negative bacterial resistance. Revista do Instituto de Medicina Tropical de São Paulo.

[24] Li J, Wang J, Yang Y, Cai P, Cao J, Cai X (2020). Etiology and antimicrobial resistance of secondary bacterial infections in patients hospitalized with COVID-19 in Wuhan, China: a retrospective analysis. Antimicrobial Resistance & Infection Control.

[25] Contou D, Claudinon A, Pajot O, Micaëlo M, Longuet Flandre P, Dubert M (2020). Bacterial and viral co-infections in patients with severe SARS-CoV-2 pneumonia admitted to a French ICU. Annals of intensive care.

[26] Bahceci I, Yildiz IE, Duran OF, Soztanaci US, Harbawi ZK, Senol FF (2022). Secondary bacterial infection rates among patients with COVID-19. Cureus.

[27] Baskaran V, Lawrence H, Lansbury LE, Webb K, Safavi S, Zainuddin NI (2021). Co-infection in critically ill patients with COVID-19: an observational cohort study from England. J Med Microbiol.

[28] Karataş M, Yaşar-Duman M, Tünger A, Çilli F, Aydemir Ş, Özenci V (2021). Secondary bacterial infections and antimicrobial resistance in COVID-19: comparative evaluation of pre-pandemic and pandemic-era, a retrospective single center study. Ann Clin Microbiol Antimicrob.

